# Multidimensional assessment of the biological effects of electronic cigarettes on lung bronchial epithelial cells

**DOI:** 10.1038/s41598-024-55140-3

**Published:** 2024-02-23

**Authors:** Meng Wang, Qing Cheng, Zehong Wu, Longjiang Fan, Linghui Zeng, Hongyu Chen

**Affiliations:** 1 Hangzhou Center for Disease Control and Prevention, Hangzhou, 31021 China; 2 Institute of Bioinformatics, Zhejiang University, Hangzhou, 310058 China; 3 RELX Science Center, Shenzhen RELX Tech. Co. Ltd., Shenzhen, 518101 China; 4 School of Medicine, Hangzhou City University, Hangzhou, 310015 China

**Keywords:** Cigarette smoke, Electronic cigarettes, Bronchial epithelial cells, Transcriptomic analysis, Mint flavor, Risk factors, Data processing

## Abstract

Cigarette smoke (CS) exposure is known to cause injury to respiratory tract epithelial cells and is a contributing factor in the development of chronic obstructive pulmonary disease and lung cancer. Electronic cigarettes (e-cigarettes) are gaining popularity as a potential substitute for conventional cigarettes due to their potential for aiding smoking cessation. However, the safety of e-cigarettes remains uncertain, and scientific evidence on this topic is still limited. In this study, we aimed to investigate the effects of CS and e-cigarette smoke (ECS) of different flavors on human lung bronchial epithelial cells. Real-time smoke exposure was carried out using an air–liquid interface system, and cell viability was assessed. RNA-Seq transcriptome analysis was performed to compare the differences between CS and ECS. The transcriptome analysis revealed a significantly higher number of differentially expressed genes in CS than in ECS. Moreover, the impact of mint-flavored e-cigarettes on cells was found to be greater than that of tobacco-flavored e-cigarettes, as evidenced by the greater number of differentially expressed genes. These findings provide a reference for future safety research on traditional cigarettes and e-cigarettes, particularly those of different flavors. The use of omics-scale methodologies has improved our ability to understand the biological effects of CS and ECS on human respiratory tract epithelial cells, which can aid in the development of novel approaches for smoking cessation and lung disease prevention.

## Introduction

Cigarette smoke (CS) is widely acknowledged as the primary cause of airway diseases, such as chronic obstructive pulmonary disease (COPD), asthma, and lung cancer^1^. Given the severe health implications, both the public and government officials have directed substantial attention toward smoking. To mitigate the harm of smoking, various “harm-reduced” tobacco products have been developed, including lower tar or nicotine content in conventional cigarettes^2^, electronic cigarettes generating nicotine aerosol rather than tobacco smoke^3^, and “heat-not-burn” devices, claiming reduced toxic chemical emissions^4^. A comprehensive understanding of the health effects of these products is vital. However, their actual harm reduction and safety remain contentious, necessitating rigorous scientific examination to inform effective public health policies and interventions for smoking-related diseases.

Historically, the focus of researching the adverse effects of cigarette smoke (CS) primarily centered on the examination of total particulate matter and gas aerosol phase extracts^5^. However, CS is a complex aerosol, comprising over 6000 identified chemical compounds^6^. In the pathogenesis of smoking-induced diseases, airway cells and tissues directly interact with the broad spectrum of reactive substances in CS, leading to inflammation^7^. To provide more precise assessments of CS-related health risks, it is imperative to evaluate the biological responses to the complete aerosol. Animal model research elucidates the harmful impact of e-cigarette aerosols on lung health, immune responses, and gene expression. Additionally, studying nicotine effects via these systems in rodents provides crucial insights into potential human health risks, aiding in formulating effective public health policies^8,9^. However, in response to the 3Rs principle, novel systems such as the In vitro air–liquid interface (ALI) models have been proposed for relevant toxicological studies^10,11^. In vitro air–liquid interface (ALI) models of respiratory tissue have evolved to evaluate genotoxicity, mutagenicity, and cellular reactions to tobacco smoke particulate matter. These advanced ALI models now closely resemble human in vivo tissue, allowing toxicological investigations with human cells^12–14^. Utilizing in vitro models to assess the toxicological effects of CS on human cells holds promise for studying smoking’s health effects. Furthermore, ALI models play a vital role in comprehending the mechanisms behind smoking-induced diseases, providing a controlled environment for researchers to explore cellular responses to specific CS constituents. Consequently, ALI models are crucial for enhancing our understanding of the health effects of CS and for formulating novel strategies to mitigate the harms associated with smoking.

In vitro models have emerged to assess the potential health risks and detrimental effects of cigarette smoke (CS), employing various cytotoxicity assays, including neutral red uptake (NRU), lactate dehydrogenase (LDH), methyl thiazolyl tetrazolium (MTT), Ames Salmonella reverse mutagenicity (AMES), and in vitro micronucleus (MN) tests^15–19^. Furthermore, in vitro models have been extensively employed in the field of electronic cigarettes. Duautoir et al. utilized the Vitrocell® VC1 smoking machine to establish an in vitro model for exposure to aerosols from heated tobacco products (HTPs), electronic cigarettes (e-Cigs), and conventional cigarette smoke. They assessed the impact on BEAS 2B cells after 1 h exposure by examining cell viability and glutathione content, among other cellular biological indicators. The findings suggested that the harm caused by HTPs might be lesser than that of conventional cigarettes but considerably greater than that of electronic cigarettes^20^. Similarly, Noël et al. employed a comparable ALI exposure system to compare the effects of electronic cigarettes with different humectant ratios and flavors on BEAS 2B cells. By measuring cell viability, intracellular and extracellular reactive oxygen species, and Nitric Oxide levels, they found that the toxicity of strawberry-flavored ENDS aerosols primarily manifested through alterations in oxidative stress levels. In contrast, the toxicity of vanilla-flavored ENDS aerosols was predominantly observed at the molecular level, involving gene dysregulation associated with biotransformation, inflammation, and oxidative stress processes^21^. Beyond these cell-based approaches, the integration of omics technologies in toxicology assessment has evolved. Transcriptomics, reflecting the genome-wide response to environmental stress at the gene level, proves to be more sensitive than individual-level indicators. Gene expression as an effect endpoint responsive to environmental toxicants often precedes organism-level survival and reproductive indicators, making it a pivotal component in environmental risk early warning research^22,23^. For instance, RNA profiling of tumors is used to predict tumor aggressiveness, understand treatment responses, and assess the risks of cell rejection^24,25^. Moreover, in the study of smoking risks, transcriptomic data from air–liquid interface (ALI) bronchial tissue cultures have offered deeper insights into the disrupted biological mechanisms by CS^26,27^. In vitro models have been employed to examine differential gene expression, revealing that e-cigarette aerosol, though less cytotoxic to human bronchial epithelial cells, induces distinct transcriptomic signatures with or without added nicotine^28^. Beyond transcriptome analysis, the perturbation of metabolism in CS-exposed tissues, global gene expression profiles, and protein alterations can be combined to identify key regulators of perturbation processes. For example, Ishikawa et al.^29^ identified that CS interfered with central carbon metabolism, induced oxidative stress, and affected epidermal growth factor receptors through multi-omics. In summary, in vitro models and omics technologies serve as potent tools for assessing the risks and harmful effects of CS on human health, allowing for the identification of potential molecular-level mechanisms of toxicity.

While electronic cigarettes have been marketed as a safer alternative to traditional tobacco, concerns regarding potential health risks associated with e-liquid chemicals, particularly flavorings, continue to rise^30^. To comprehensively understand the toxicological mechanisms of electronic cigarette smoke (ECS) and evaluate the safety implications of different ECS flavors, our study encompassed immediate and 4 h post-exposure comparisons to evaluate the time-dependent effects of ECS exposure on cell viability. Additionally, we examined varying dilution levels of ECS to discern any dosage-related impacts on cellular health. These assessments were integral in understanding the nuanced impacts of electronic cigarette smoke (ECS) exposure on human cell viability. We investigated the impact of different nicotine concentrations in electronic cigarette liquids on human cell responses, aiming to identify potential variations in cellular health outcomes. This comprehensive approach allowed us to scrutinize the effects of varying nicotine levels present in ECS on cellular health. Furthermore, comparative experiments were conducted between menthol and tobacco-flavored e-liquids to discern potential differential effects on cellular health. Through these comparisons, we sought to uncover any distinct impacts of different flavorings commonly found in electronic cigarette liquids. In addition, we rigorously scrutinized the disparate impacts of different components (solvents and nicotine) in e-liquids through comparative assessments, evaluating their potential effects on cellular health. This thorough investigation aimed to dissect the individual contributions of these components towards cellular responses upon exposure. Employing a range of comparisons and methodologies, our study aimed to provide a comprehensive understanding of the potential health implications associated with diverse electronic cigarette flavors and components. These findings shed light on the safety considerations of ECS exposure and its potential implications for human health.

## Methods and materials

### Cell culture

The human bronchial epithelial cell line BEAS-2B cells were obtained from the Shao laboratory, which is affiliated with the Institute of Medicine at Zhejiang University in Hangzhou, China. BEAS-2B cells were cultured in DMEM basic nutrient solution supplemented with 10% fetal calf serum, 2 mM L-glutamine, and 100 units/ml Penicillin–Streptomycin, and were maintained in a CO_2_ incubator at 37 °C with 5% CO_2_ (v/v) (Thermo scientific, China). The cells were passaged when they reached 80%-90% confluence. The DMEM basic nutrient solution was purchased from Gibco, a brand under Thermo Fisher Scientific in China, while the fetal calf serum was obtained from GR, a subsidiary of Gibco in New York, USA. L-glutamine was sourced from AR, a manufacturer based in Beijing, China, while the Penicillin–Streptomycin was obtained from HyClone, a subsidiary of GE Healthcare Life Sciences, now part of Cytiva, in Beijing, China.

### Experimental cigarettes

Two flavors of e-cigarettes, including the tobacco flavor (ECS-T) and mint flavor (ECS-M) variants (RELX), were used for our study. Each type of e-cigarette was available in two different nicotine concentrations, namely, a no-nicotine variant and a 4% nicotine variant. The nicotine and tar contents of the e-cigarettes are summarized in Table 1. We also selected 3R4F reference cigarettes from the University of Kentucky in Lexington, KY, USA for our exposure experiments. The traditional cigarettes were conditioned for at least 48 h at 22 ± 1 °C and 60 ± 3% relative humidity prior to use in the experiments. By utilizing the 3R4F reference cigarette along with electronic cigarettes with varying nicotine and tar levels, as well as diverse e-cigarette flavors, our study aimed to investigate the potential risks posed by various types of electronic cigarettes on bronchial epithelial cells in comparison to traditional combustible cigarettes.

**Table 1:** Summary of smoke exposure experiments in this study.

Experiments	Cigarette type	Sample code	Tar (mg/cig)	Nicotine (mg/cig)	Dilution times	Sampling time (h) after exposure
Optimal concentration	Electronic cigarette	T4	–	40	1/5, 1/10, 1/30	0, 4
Transcriptomic profiling: reference conventional cigarette versus electronic cigarette with different flavors	Air	Air	–	–	–	4
	Reference cigarette	3R4F	9.4	0.73	1/500	4
	Electronic cigarette	K0	–	0	1/5	4
	Electronic cigarette	K4	–	40	1/5	4
	Electronic cigarette	T0	–	0	1/5	4
	Electronic cigarette	T4	–	40	1/5	4
	Electronic cigarette	M0	–	0	1/5	4
	Electronic cigarette	M4	–	40	1/5	4

K0 represents an e-cigarette containing only solvent, with the main ingredients being glycerol and propylene glycol. K4 represents an e-cigarette with the addition of 4% nicotine to the e-liquid, in addition to glycerol and propylene glycol. T0 represents an e-cigarette with the addition of tobacco-flavored spices to the e-liquid, but without nicotine. M0 represents an e-cigarette with the addition of mint-flavored spices to the e-liquid, but without nicotine. M4 represents mint flavored electronic cigarette with 4% nicotine, and T4 represents tobacco flavored electronic cigarette with 4% nicotine.

### Air–liquid interface model for in vitro cell exposure treatment to aerosols

In order to conduct exposure experiments with CS and ECS, the BEAS-2B cells were seeded onto transwell inserts with a 3 μm polycarbonate membrane at a density of 2.0 × 10^4^ cells/cm^2^, and an ALI was established by removing the medium from the apical surface, leaving the basal surface of the cells exposed to medium. The BEAS-2B cells were then exposed to air, CS, and ECS using a Borgwaldt RM20S rotary syringe smoking machine, specifically designed for in vitro biological toxicity assessments of CS^31^. To avoid potential exposure to CS components in the nutrient solution due to aerosol sedimentation, a peristaltic pump was used to replace the nutrient solution at a flow rate of 3 ml/min. The CS and ECS were diluted with laboratory air to the appropriate concentrations (Table 1) before being used to expose the cells maintained at the ALI in exposure chambers housed at 37 °C. The puffing regimen was set according to Health Canada guidelines, which involved a 55 ml puff drawn over 1 s every 30 s period. Prior to sampling, all cells were cultured in a CO_2_ incubator for 4 h. Cytotoxicity assays and RNA extraction were subsequently performed. Negative control groups were achieved with air-exposed cells under the same protocol as the CS and ECS-exposed cells.

### Nicotine content determination

To determine the concentration of nicotine in cigarette smoke (CS) and electronic cigarette smoke (ECS). The method involved the use of Cambridge filters to collect the smoke samples, which were then immediately extracted with 20 ml of absolute ethanol. The nicotine content was analyzed using ultra-performance liquid chromatography (UPLC) with an ACQUITY UPLC HSS T3 analytical column. The mobile phase was a mixture of 10 mM ammonium acetate and acetonitrile, which was pumped at a flow rate of 0.3 ml/min. The optimal gradient elution was achieved by varying the mobile phase from 80:20 A-B to 10:90 A-B, and then back to 80:20 A-B. The column temperature was set to 40 ℃, and the injection volume was 0.5 μl. Detection of the nicotine content was performed using UV chromatograms at a wavelength of 260 nm. The nicotine content was quantified using an external standard method, and each type of cigarette was tested six times to obtain an accurate representation of the results.

### Trans-endothelial electrical resistance measurement

The electrical parameter, trans-endothelial electrical resistance (TEER), was utilized to evaluate the integrity of the cellular barriers in vitro following exposure to CS. TEER was measured using a Millicell-ERS-2 (Millipore, Billerica, MA, US) volt-ohm-meter equipped with a STX01 chopstick electrode (Millipore, Billerica, MA, US). Three replicate measurements were taken for each well, and three wells were tested per experimental run. The obtained values were adjusted with air controls and then multiplied by the effective membrane area in cm^2^ (1.12 cm^2^ for 12-well transwell inserts) to obtain the final result in Ω cm^2^. TEER measurements allowed for the assessment of cell layer integrity and toxicity following exposure to CS, providing an objective measure for the suitability of the cellular model. To evaluate the impact of various treatments on cellular Trans Epithelial Electrical Resistance (TEER) values, we utilized the control group as a baseline. We computed the differences in TEER values between different treatment groups and the control group to depict the extent of influence on cellular membrane integrity.

### Neutral red dye uptake assay

The evaluation of cytotoxicity in tobacco product testing is typically carried out using the neutral red uptake (NRU) assay. This method involves the use of an acidotropic stain, neutral red, which is sequestered by lysosomes. When lysosomal membranes are compromised by toxic substances, the uptake and binding of the dye is decreased. In the current study, BEAS-2B cell samples were treated with nutrient medium containing 50 µg/ml neutral red for a period of 3 h. The dye was then extracted, and the absorbance was measured at 540 nm using a spectrophotometer (Synergy H1, USA). Cell viability was calculated by determining the ratio of absorbance in the treatment group relative to that in the control group. To ensure the reliability of the results, each experiment run was carried out in triplicate using three wells.

### Transcriptomic profiling of BEAS 2B cells under smoke exposure

RNA was isolated from the cell samples using the RNeasy mini kit, as per the manufacturer's instructions. The quantity of RNA was determined using a NanoDrop ND1000, and samples with a RIN number greater than 8 were used for subsequent analyses. Paired-end sequencing was carried out on an Illumina Hiseq 4000 for RNA-seq, with triplicates performed for each treatment and control. A total of 30 samples were generated in accordance with the experimental design, and all sequencing data was submitted to NGDC with the accession number PRJCA016303. Quality control metrics for initial reads were generated using FASTQC^32^, and low-quality read ends were trimmed using trimmomatic^33^. HISAT aligner was used with default parameters to align the RNA-seq reads to the human genome (GRCh38)^34^. Differential expression genes were analyzed using the edgeR package^35^, with genes demonstrating an adjusted *p* value < 0.05 and fold change (FC) > 2 or < − 2 considered statistically significant. The significantly modulated genes were further analyzed for GO classification using the Clusterprofiler package^36^.

## Results

### Response of cells exposed to e-cigarettes varied depending on the concentration and duration of recovery

**Figure 1:**
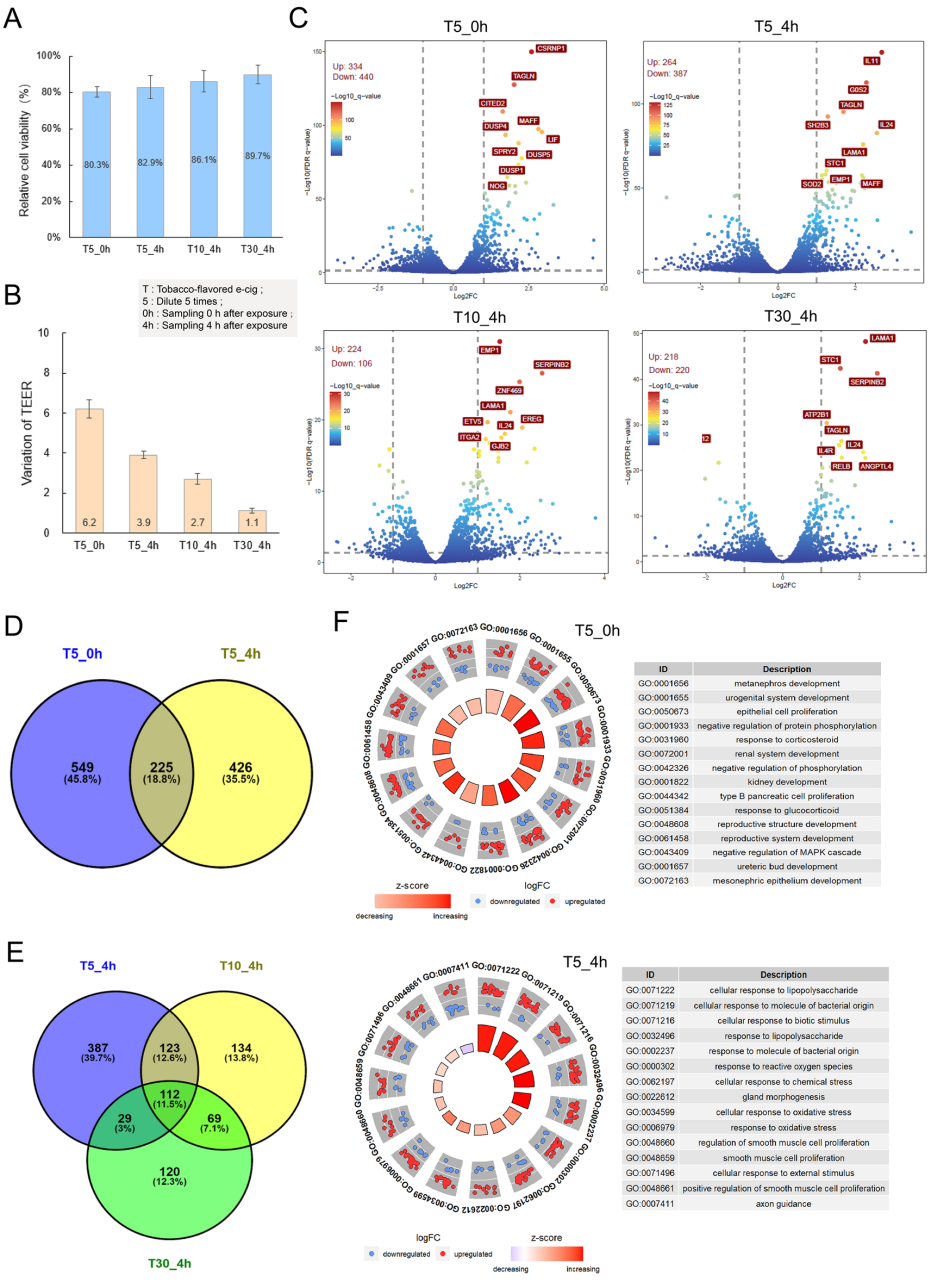
The effect of electronic cigarette aerosol treatment on cells varies with different recovery times and dilution factors (T5_0h represents recovery at 0 h after fivefold dilution treatment of 4% nicotine tobacco-flavored e-cigarette; T5_4h represents recovery at 4 h after the same e-cigarette treatment; T10_4h represents recovery at 4 h after tenfold dilution treatment of the same e-cigarette; T30_4h represents recovery at 4 h after 30-fold dilution treatment of the same e-cigarette.). (**A**) The changes in cell viability under different treatments. (**B**) The degree of change in TEER values under different treatments. (**C**) The volcano plot shows the changes in the transcriptome under different treatments. (**D**) The Venn diagram illustrates the similarities and differences in transcriptome changes at different recovery time points. (**E**) The Venn diagram illustrates the degree of similarities and differences in transcriptome changes under different dilution factors of the aerosol treatment. (**F**) GO enrichment (BP level) circle plots for the top five most significant GO categories with positive and negative z-score under electronic cigarette aerosol treatment with two time points. The outer circle shows the relative fold change for each significant RNA feature compared to air contributing to the GO term, where blue dots represent down-regulated RNA features and red dots represent up-regulated features. The inner quadrants are colored based on the z-score, and its surface is a function of the enriched q-value. The larger the surface, the lower the q-value. The Z-score color scale represents the number of up-regulated genes for a given GO term minus the number of down-regulated genes divided by the square root of the total count. The related table shows the GO item ID and function.

To determine the exposure duration and concentration of e-cigarettes on BEAS 2B cells, experiments were conducted using commonly available e-cigarettes (with nicotine content determined as 4% via UPLC). BEAS 2B cells were exposed to e-cigarette aerosol for 1 h, and assessments, including Neutral Red staining, transepithelial electrical resistance (TEER) measurements, and RNA collection, were conducted at both 0 h and 4 h. Previous research indicated sustained effects on cells following exposure to 3R4F cigarette smoke^31^. After one hour of e-cigarette aerosol treatment (diluted fivefold), BEAS 2B cell viability remained at approximately 80%. Following four hours of recovery, cell viability increased slightly without significant differences (Fig. 1A). Notably, the change in TEER values after four hours showed minor fluctuations, suggesting partial recovery in cell growth (Fig. 1B). These results suggest that e-cigarette aerosol exposure did not exacerbate the cellular impact.

Furthermore, transcriptome analysis was conducted on samples recovered at different time points, revealing differential gene expression patterns. T5_0h identified 774 differentially expressed genes, consisting of 334 upregulated and 440 downregulated genes, surpassing the numbers in T5_4h, which featured 264 upregulated and 387 downregulated genes (Fig. 1C). A Venn diagram analysis showed that only 225 genes were common between the two sets of differentially expressed genes, indicating potential functional disparities (Fig. 1D). Subsequent GO enrichment analysis was based on these identified differentially expressed genes (Fig. 1F). The T5_0h group exhibited enrichment in pathways associated with growth and development, such as developmental pathways and epithelial cell proliferation, suggesting an impact of e-cigarette aerosol treatment on cell growth. Consistent with previous research indicating a reduction in nasal epithelial cell growth due to e-cigarettes^37^. In the T5_4h group, the enriched pathways shifted towards response-related pathways, including cellular response to lipopolysaccharide, response to biotic stimulus, response to external stimulus, and wound healing regulation. This suggests that, after four hours of recovery, cells initiated responses and repair mechanisms following e-cigarette aerosol treatment. To ensure consistency with 3R4F reference cigarettes and for subsequent experiments, we selected the four-hour recovery time point as the detection time for all future investigations.

In addition, we analyzed whether different dilution factors had varying effects of e-cigarette aerosol on cells. We considered three dilution factors (1/5, 1/10, 1/30). From a cell viability perspective, as the dilution fold increased, cell viability also increased, surpassing 80%, with no significant differences observed (Fig. 1A). The change in TEER values indicated that e-cigarette aerosol diluted 30 times had nearly no effect on cell transmembrane resistance, suggesting a dose–response effect similar to previous findings in the 3R4F study on e-cigarette aerosol treatment. Further transcriptome analysis revealed 330 differentially expressed genes in the tenfold dilution group (T10_4h) and 438 genes in the 30-fold dilution group (T30_4h). Only 112 genes were common among the three groups, suggesting a clear dose–response relationship (Fig. 1E). GO enrichment analysis showed that T30_4h enriched pathways were similar to T10_4h, including extracellular structure organization and development-related pathways (Fig. S1), reinforcing the presence of a discernible dose–effect relationship in e-cigarette aerosol treatment.

### E-cigarettes exhibit lower biological impact compared to cigarettes

**Figure 2:**
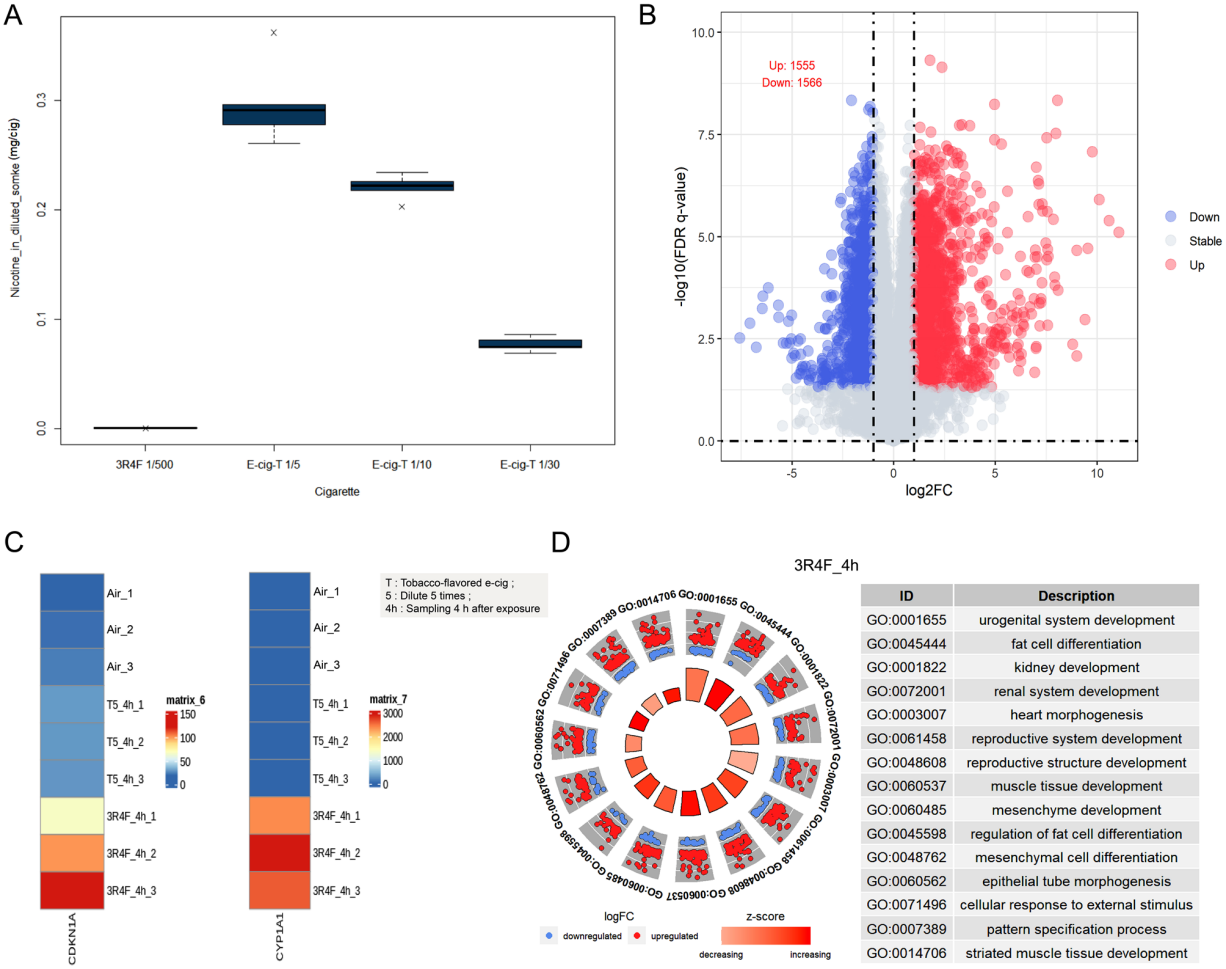
(**A**) The box plot displays the nicotine content in cigarette smoke (CS) and e-cigarette aerosol (ECS-T) under different dilution factors. The horizontal line in the middle of the box represents the mean value of the data. (**B**) The volcano plot shows the transcriptome changes in cells after treatment with 3R4F cigarette smoke. Blue points indicate down-regulated genes, while red points indicate up-regulated genes. (**C**) The figure shows the expression levels of CDKN1A and CYP1A1, two biomarkers of smoke exposure, in BEAS 2B cells under different smoke exposures. (**D**) GO enrichment circle plots for the top five most significant GO categories with positive and negative z-score under 3R4F treatment.

Electronic cigarettes (e-cigarettes) are considered a safer alternative to traditional tobacco cigarettes, which produce cigarette smoke. To compare the effects of e-cigarette vapor and 3R4F cigarette smoke on BEAS 2B cells, previous studies indicated that when 3R4F cigarette smoke was diluted 500 times, cell viability reached approximately 80%, comparable to cell vitality observed when ECS was diluted five fold^31^. In this study, a 1/500 dilution factor was used for 3R4F cigarette treatment. Nicotine content in different cigarette smoke types was quantified using ultra-high performance liquid chromatography (UPLC). Results showed much lower nicotine content in 500-fold diluted cigarette smoke compared to e-cigarette smoke across different dilution factors. The nicotine content in e-cigarette smoke decreased with increasing dilution factor (Fig. 2A). These results suggest that nicotine may not be the primary factor influencing cellular activity.

To investigate the effects of CS and ECS on cell gene expression, RNA-Seq analysis was conducted using total RNA from cells treated with 1/5 ECS and 1/500 CS. Three replicates were performed for each treatment and control, generating an average of 7 Gb paired-end RNA-seq data per sample. Over 95% of reads successfully aligned to the human reference genome (GRCh38). In the CS group, 3121 genes were identified, with 1555 upregulated and 1566 downregulated genes (Fig. 2B). In contrast, only 774 differentially expressed genes were found in the ECS group, with 334 upregulated and 440 downregulated genes (Fig. 1C). These included smoke exposure biomarker genes like cyclin-dependent kinase inhibitor 1A (CDKN1A) and cytochrome P-450 s (e.g., CYP1A1 involved in polycyclic aromatic hydrocarbon bioactivation). The heatmap illustrated that cigarette smoke treatment induced these genes more strongly than e-cigarette vapor treatment (Fig. 2C). The differential gene expression pattern aligned with previous studies on HBE cells’ response to cigarette smoke^28^. GO pathway analysis highlighted differences in gene expression between CS and ECS-treated cells. 3R4F cigarette treatment enriched pathways related to growth, development, and responses akin to e-cigarette smoke, including fat cell differentiation, reproductive structure development, and cellular response to chemical stress. Cigarette treatment exhibited a higher proportion of genes and pathways in these processes compared to e-cigarettes. Furthermore, exposure to cigarette smoke led to the upregulation of pathways associated with inflammation, including the stress-activated MAPK cascade, the regulation of apoptotic signaling pathways, stress-activated protein kinase signaling cascades, and MAP kinase phosphatase activity (Fig. 2D). Wang's study reported that acute exposure to conventional cigarette smoke (SC) significantly impacts cellular responses and gene expression profiles when compared to exposure to electronic cigarette smoke (ECS)^38^. To sum up, despite e-cigarettes containing higher nicotine levels, they exhibit significantly lower cytotoxicity towards BEAS-2B cells when compared to traditional cigarettes.

### Solvent was identified as one of the main causes of cellular effects resulting from exposure to electronic cigarettes

Electronic cigarette liquid typically consists of a mixture of propylene glycol, vegetable glycerin, flavorings, and nicotine (although some e-liquids are nicotine-free). Propylene glycol and vegetable glycerin are utilized to generate aerosols and are considered safe food ingredients according to the regulations set forth by the United States Food and Drug Administration (FDA). Flavorings can vary widely, ranging from fruit and dessert flavors to more unique options such as tobacco and mint, among others. Nicotine is an optional component with its concentration varying according to user preferences. While these solvents are generally regarded as food-safe, their impact on cells when aerosolized and inhaled is still under investigation. Some studies have indicated that nicotine plays a notable role in causing negative effects, with solvents alone not resulting in any adverse outcomes. However, contrasting research has proposed that nicotine is not linked to adverse effects, while emphasizing that solvents and flavorings are the primary constituents responsible for eliciting significant harmful effects on cells or animals^39^.

**Figure 3:**
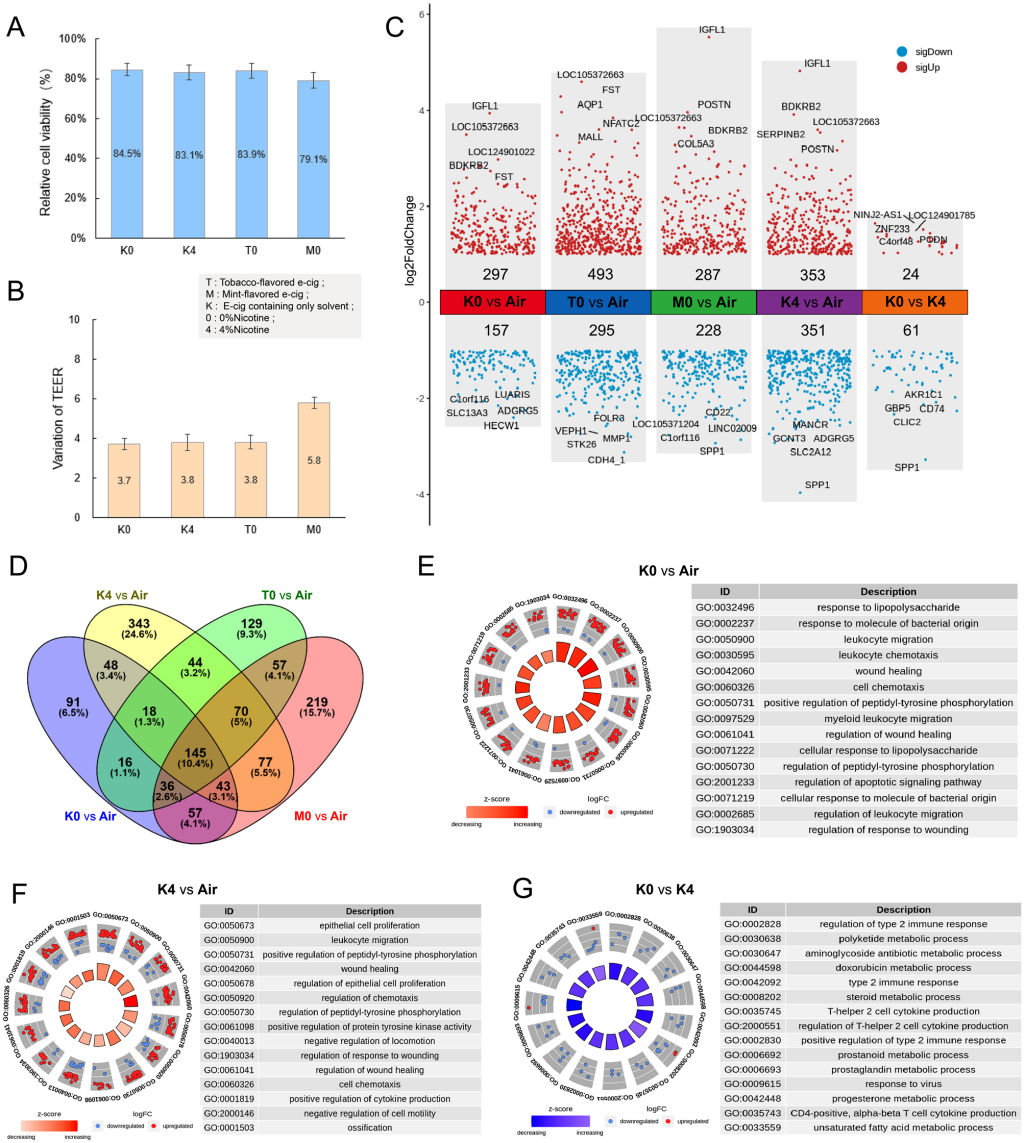
The effects of different types of electronic cigarette aerosol treatments on BEAS 2B cells. K0 represents an e-cigarette containing only solvent, with the main ingredients being glycerol and propylene glycol. K4 represents an e-cigarette with the addition of 4% nicotine to the e-liquid, in addition to glycerol and propylene glycol. T0 represents an e-cigarette with the addition of tobacco-flavored spices to the e-liquid, but without nicotine. M0 represents an e-cigarette with the addition of mint-flavored spices to the e-liquid, but without nicotine. (**A**) The effects of different types of electronic cigarette treatments on BEAS 2B cell viability. (**B**) The degree of TEER value changes under different types of electronic cigarette treatments. (**C**) Differential transcriptomic changes of BEAS 2B cells under different types of electronic cigarette (e-cigarette) treatments, with upregulated genes represented by red dots and downregulated genes represented by blue dots. (**D**) The Venn diagram shows the degree of similarity and difference in differentially expressed genes among different types of e-cigarette treatments. (**E**–**F**) GO enrichment (BP level) circle plots for the top five most significant GO categories with positive and negative z-score under K0 and K4 treatment. (**G**) GO enrichment analysis of differentially expressed genes between K0 and K4 treatment.

Therefore, our study aimed to compare the effects of e-cigarettes that included solvents (consisting solely of glycerin and propylene glycol) with those containing 4% nicotine. From the perspective of cell viability, cell survival rates remained around 80% in different e-cigarette aerosol treatments (Fig. 3A), with a similar degree of change in TEER values, except for a more significant impact observed in the M0 group (Fig. 3B). To further compare the differences between these e-cigarette groups, transcriptome differential analysis was performed (Fig. 3C). Even with only solvents, the K0 group led to differential expression of 454 genes, and the number of differentially expressed genes increased when flavorings and nicotine were added. Differential genes enriched in the K0 group were primarily concentrated in pathways related to stimuli response, such as response to lipopolysaccharide, wound healing, and wound healing regulation, suggesting that even solvents exert a significant influence on cells (Fig. 3E). Similarly, the K4 group with added nicotine exhibited similar results in enrichment analysis (Fig. 3F). Furthermore, a comparative analysis between the K0 and K4 groups revealed 85 differentially expressed genes, including 24 upregulated genes and 61 downregulated genes. Functional enrichment analysis revealed significant enrichment in immune regulation and metabolic processes, such as regulation of type 2 immune response, type 2 immune response, and polyketone metabolism, among others (Fig. 3G). Additionally, when comparing the effects of adding different flavors, the addition of mint flavor (M0 vs Air) resulted in the highest number of differentially expressed genes (Fig. 3D), indicating its impact surpasses that of tobacco flavor, warranting further investigation.

### Mint flavor shows stronger impact than tobacco flavor

**Figure 4:**
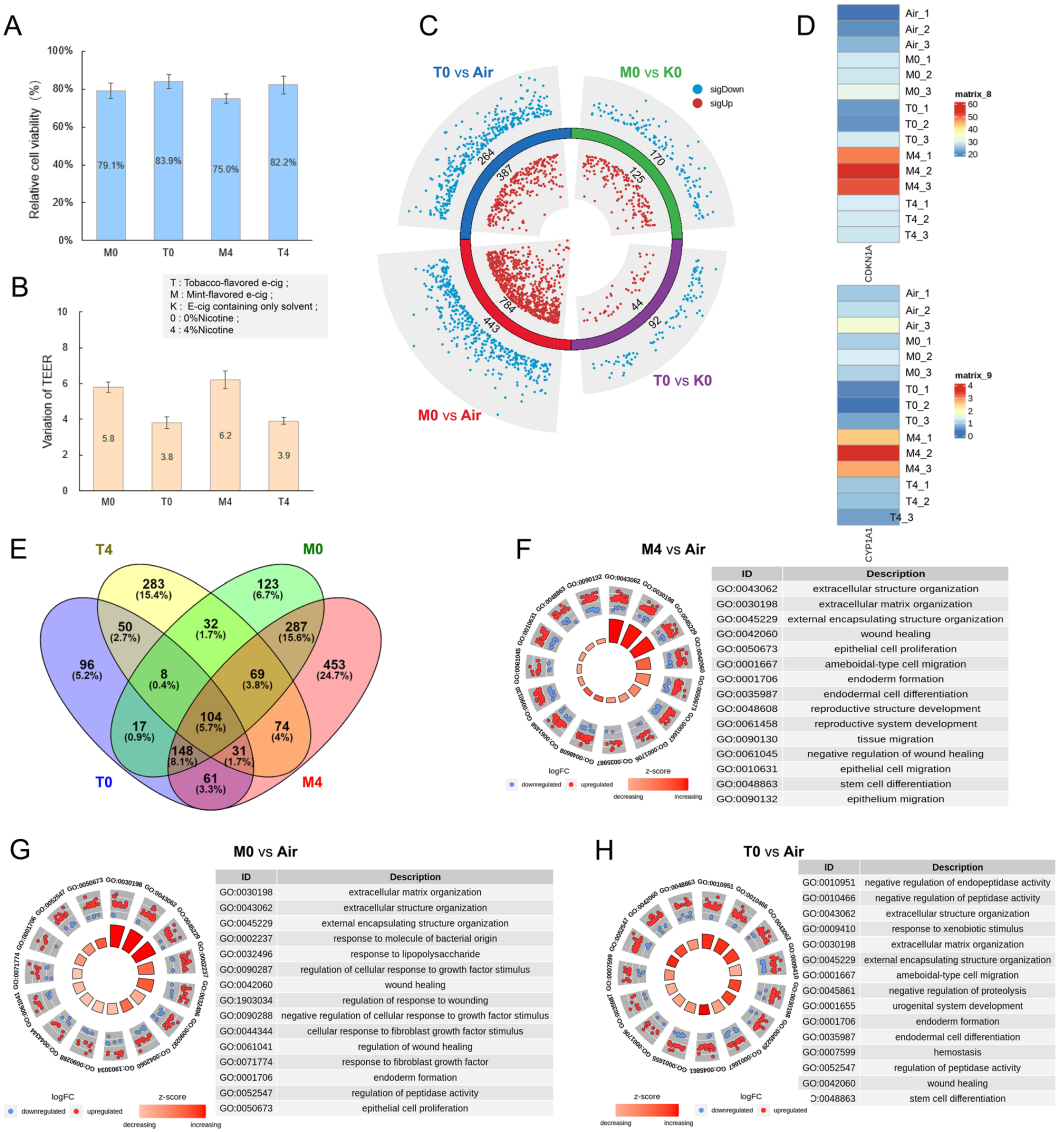
The effects of different flavored electronic cigarette treatments on BEAS 2B cells. M0 represents nicotine-free mint flavored electronic cigarette, T0 represents nicotine-free tobacco flavored electronic cigarette, M4 represents mint flavored electronic cigarette with 4% nicotine, and T4 represents tobacco flavored electronic cigarette with 4% nicotine. (**A**) The effect of different flavored electronic cigarette treatments on cell viability. (**B**) The degree of TEER changes under different flavored e-cigarette treatments. (**C**) Differential gene expression analysis of BEAS 2B cells under different e-cigarette flavor treatments. Red dots indicate upregulated genes, blue dots indicate downregulated genes, and the sharper and wider the sector, the greater the degree of gene expression change. (**D**) This figure displays the expression levels of the biomarkers of smoke exposure, CDKN1A and CYP1A1, in BEAS 2B cells exposed to aerosols from different flavored e-cigarettes. (**E**) The Venn diagram shows the degree of overlap of differentially expressed genes under different treatments. (**F**) GO enrichment (BP level) circle plots for the top five most significant GO categories with positive and negative z-score under M4 treatment. (**G**) GO enrichment analysis of differentially expressed genes between Air and M0 treatment. (**H**) GO enrichment analysis of differentially expressed genes between Air and T0 treatment.

Recent federal regulations have banned the sale of flavored e-cigarettes, with the exception of tobacco or mint flavors^40^. Furthermore, the European Union and select Asian nations have also implemented prohibitions on specific flavored e-cigarette products, encompassing fruit, sweet, and mint varieties. Diverse e-cigarette flavors result from varying additives and formulations employed by distinct brands and manufacturers, giving rise to a spectrum of tastes and mouthfeel experiences. In our study, we have initially found that mint-flavored e-cigarettes have a more significant impact on cells compared to their tobacco-flavored counterparts. Demonstrated through assessments of cell viability and Trans-Epithelial Electrical Resistance (TEER) values, mint-flavored e-cigarettes exhibit a more substantial effect on cellular health, irrespective of the presence of nicotine (Fig. 4A,B). These findings are consistently mirrored at the transcriptomic level, with the number of differentially expressed genes induced by mint-flavored e-cigarettes significantly surpassing those elicited by tobacco-flavored variants, irrespective of nicotine content (Figs. 3C, 4C). These differentially expressed genes are primarily associated with pathways related to cell growth, development, and response, encompassing extracellular structure organization, wound healing, and epithelial cell proliferation (Fig. 4F). Furthermore, it is noteworthy that electronic cigarettes with different flavors exhibit relatively fewer commonly differentially expressed genes, indicating that various flavored electronic cigarettes exert some degree of specificity in their effects on cells (Fig. 4E).

We further investigated biomarker genes associated with exposure to e-cigarette aerosols. The heatmap representation illustrates that mint-flavored e-cigarette treatment elicits higher expression of these genes in comparison to tobacco-flavored treatment, irrespective of the presence of nicotine (Fig. 4D). Additional comparisons were conducted between nicotine-free mint (M0) and tobacco-flavored e-cigarettes (T0) and a solvent control (K0), demonstrating that mint flavor induces a greater number of differentially expressed genes than tobacco flavor (Fig. 4C). Compared to tobacco-flavored e-cigarettes (T0), mint-flavored (M0) e-cigarettes exhibit a greater enrichment of pathways related to wound healing and its response (Fig. 4G,H). The pathways influenced by mint flavor primarily relate to the regulation of cell growth and important developmental processes. In contrast, tobacco flavor predominantly affects only three pathways: alcohol dehydrogenase activity, aldo-keto reductase activity, and bile acid binding (Fig. S2). Another study also observed increased inflammation in BEAS 2B cells following exposure to mint^41^. Notably, these findings are consistent with the results of our own research. In summary, the collective results consistently support the assertion that mint-flavored e-cigarettes exert a more substantial impact on cellular physiology in comparison to their tobacco-flavored counterparts.

## Discussion

Electronic cigarettes have gained popularity as an alternative to traditional cigarettes, with their usage on the rise globally. Nonetheless, ongoing debates surround the potential health implications of electronic cigarette use. This study delves into the biological impact of electronic cigarettes on lung bronchial epithelial cells, with a particular focus on variables such as exposure concentration, duration, flavorings, and solvents. Our findings underscore the variability in cellular responses to e-cigarette exposure based on concentration and recovery time. At lower concentrations, e-cigarettes exhibit minimal to no adverse effects on cell viability and metabolic activity, whereas higher concentrations lead to a notable decline in both. This indicates the pivotal role of e-cigarette aerosol concentration in determining its biological effects on lung bronchial epithelial cells. Additionally, the duration of post-exposure recovery emerges as a significant factor, as cells exhibit the capacity for recuperation after a 4 h incubation period. When comparing the biological impact of e-cigarettes to that of traditional cigarettes, our investigation reveals a lesser biological impact associated with e-cigarettes. This observation is significant, suggesting that e-cigarettes might present a safer alternative to traditional cigarettes in terms of their influence on lung bronchial epithelial cells^42^. However, this does not unequivocally establish the absolute safety of e-cigarettes, as further research is imperative to comprehensively comprehend their long-term consequences on human health.

Furthermore, our study identifies the solvent as the primary culprit for cellular effects resulting from e-cigarette exposure. Similarly, Werley et al.^43^ conducted a 90 days study using adolescent rats to investigate the primary substances responsible for toxicity in e-cigarettes, specifically whether it was the solvents, nicotine, or flavorings. The research findings revealed that propylene glycol and glycerol, when individually exposed, exhibited the highest exposure-related pulmonary effects. This study suggests that the solvents in e-liquids can have detrimental effects when inhaled. This finding accentuates the necessity for further exploration into the potential health effects of solvents used in e-cigarettes. It also encourages e-cigarette manufacturers to explore alternative, less harmful solvents that are more conducive to human health. Lastly, our investigation reveals that mint-flavored e-cigarettes exhibit a more pronounced impact on lung bronchial epithelial cells when compared to tobacco-flavored ones. This is an intriguing discovery, suggesting that the flavorings employed in e-cigarettes may influence their biological effects on human health. The cooling effect attributed to mint flavor on the airways may contribute to its more potent impact. Rowell et al.^44^ conducted experiments examining the impact of 13 distinct flavored e-liquids on a human lung epithelial cell line, employing a biologically relevant dosing range. Their findings revealed a dose-dependent decline in cell viability. Additionally, their study identified that certain flavorings, such as mint, produced more detrimental effects compared to others. Likewise, in Omaiye et al.^45^ study, it was observed that exposure to mint-flavored e-cigarettes had a toxic effect on BEAS-2B cells. However, a comprehensive examination is needed to fully understand the biological impacts of different e-cigarette flavors on lung bronchial epithelial cells.

In summary, our study provides crucial insights into the multifaceted assessment of the biological effects of electronic cigarettes on lung bronchial epithelial cells. It underscores the importance of variables such as exposure concentration, duration, flavorings, and solvents in determining their impact on human health. Additionally, our comparative analysis with traditional cigarettes highlights the potential for e-cigarettes to represent a safer alternative, while underscoring the necessity for continued research into their long-term effects. These findings bear significant implications for public health and regulatory policies. Given the potential health risks associated with e-cigarette usage^37^, it is imperative to maintain vigilance and regulation in the sale and use of these products. This may involve imposing restrictions on e-cigarette aerosol concentration, advocating for the utilization of less harmful solvents, and governing the marketing and sale of flavored e-cigarettes. Furthermore, comprehensive research is vital to fully comprehend the potential health risks associated with e-cigarettes, especially in terms of their long-term effects on human health.

## Conclusions

In conclusion, our study provides vital insights into the biological impact of electronic cigarettes on lung bronchial epithelial cells. While suggesting that e-cigarettes may represent a safer alternative to traditional cigarettes, it underscores the necessity for continued research to fully understand their potential health risks. As the use of e-cigarettes continues to surge, it remains imperative to monitor and regulate these products to ensure the safety of users and the broader public.

### Supplementary Information

Supplementary Legends.

Supplementary Figure S1.

Supplementary Figure S2.

## Data Availability

The RNAseq data are publicly available in National Genomics Data Center database, accession number PRJCA016303.
