# Supplementary material for: Multidimensional assessment of the biological effects of electronic cigarettes on lung bronchial epithelial cells
Source: Sci Rep. 2024 Feb 23;14:4445. doi: 10.1038/s41598-024-55140-3 (PMC10891173; doi:10.1038/s41598-024-55140-3)
Supplement: Supplementary file 1 — Supplementary Legends. [file 41598_2024_55140_MOESM1_ESM.docx]

**Lengends of supplmentary figures**

**Figure S1** GO enrichment circle plots for the top five most significant GO categories with positive and negative z-score under electronic cigarette aerosol treatment with two different dilution factors. T10_4h represents recovery at 4 hours after 10-fold dilution treatment of the same e-cigarette; T30_4h represents recovery at 4 hours after 30-fold dilution treatment of the same e-cigarette.

**Figure S2** GO enrichment circle plots for the top five most significant GO categories with positive and negative z-score under electronic cigarette aerosol treatment with two flavors. M0 represents nicotine-free mint flavored electronic cigarette, T0 represents nicotine-free tobacco flavored electronic cigarette, K0 represents an e-cigarette containing only solvent.
