# Supplementary figures and images for: Multidimensional assessment of the biological effects of electronic cigarettes on lung bronchial epithelial cells
Source: Sci Rep. 2024 Feb 23;14:4445. doi: 10.1038/s41598-024-55140-3 (PMC10891173; doi:10.1038/s41598-024-55140-3)

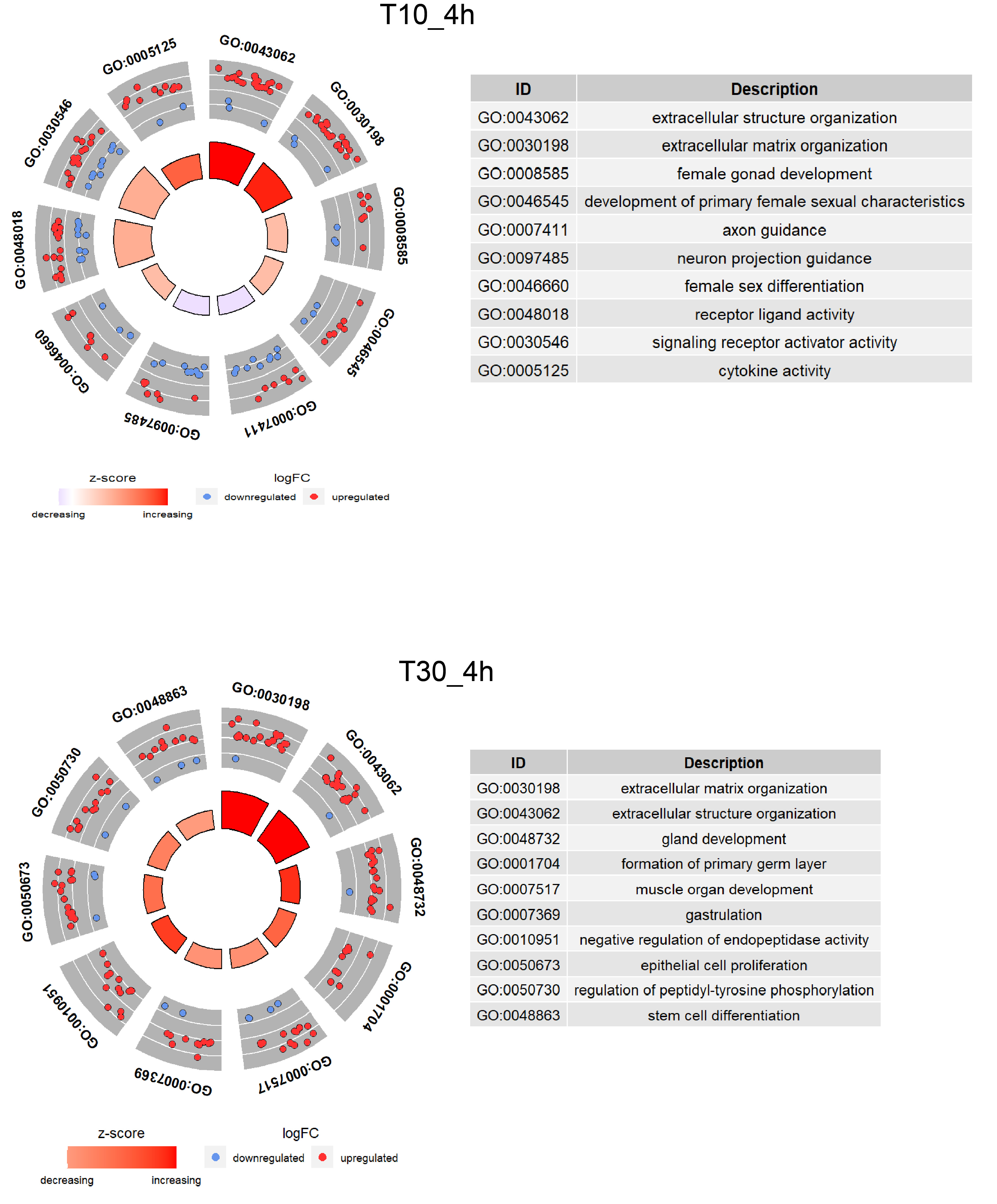

Supplement: Supplementary file 2 — Supplementary Figure S1. [file 41598_2024_55140_MOESM2_ESM.jpg]

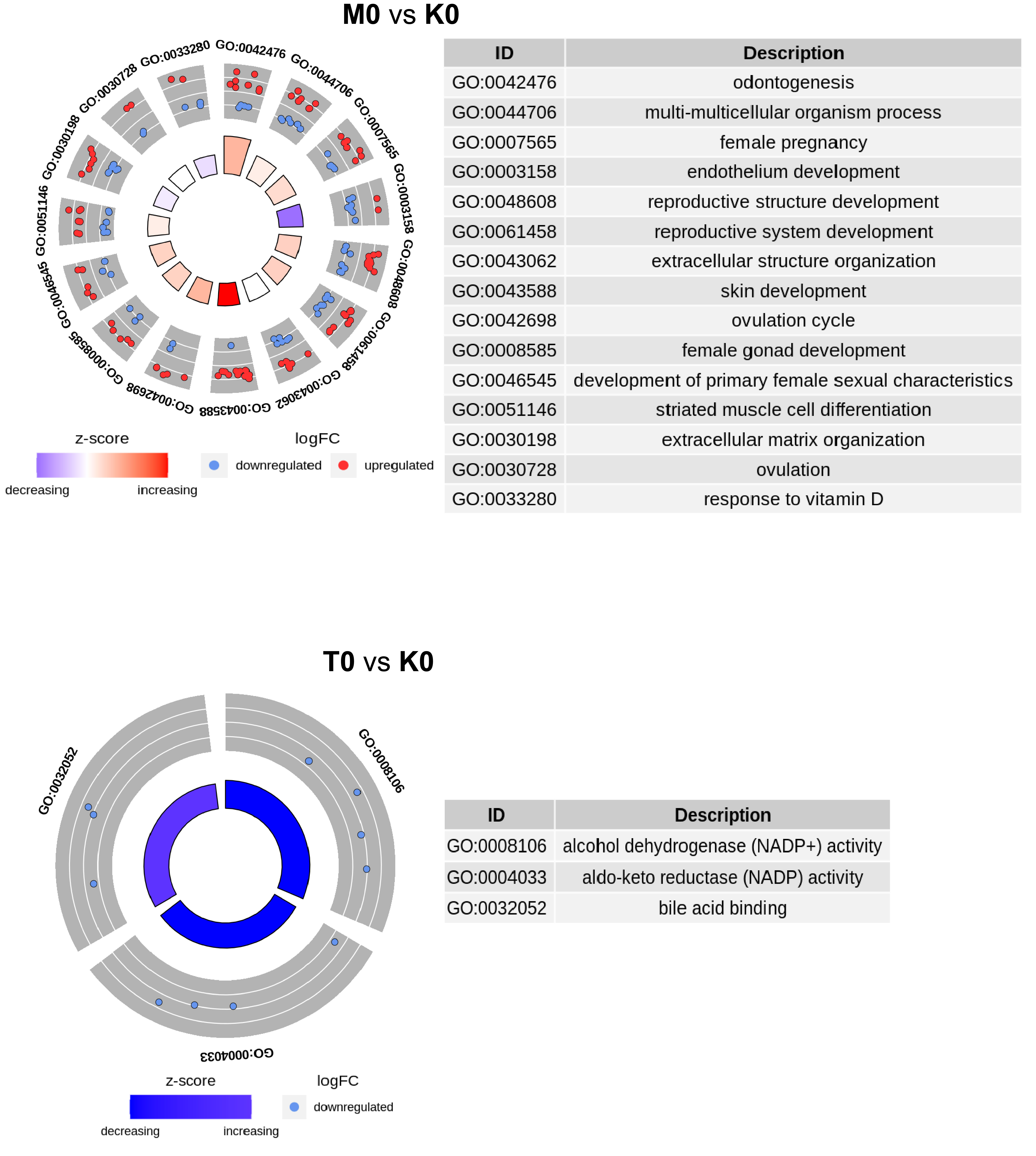

Supplement: Supplementary file 3 — Supplementary Figure S2. [file 41598_2024_55140_MOESM3_ESM.jpg]
